# From motivation to communicative readiness in blended CSL: academic emotions and academic self-efficacy as theory-specified indirect pathways

**DOI:** 10.3389/fpsyg.2026.1880186

**Published:** 2026-09-03

**Authors:** Xinying Lyu

**Affiliations:** College of International Education, China University of Petroleum, Beijing, China

**Keywords:** academic emotions, academic self-efficacy, blended CSL learning, Chinese as a second language, L2 willingness to communicate, learning motivation, structural equation modeling

## Abstract

Many learners of Chinese can complete classroom tasks and demonstrate formal knowledge, yet remain reluctant to use Chinese when communication becomes socially visible. This study examined how learning motivation is associated with L2 willingness to communicate (WTC) among international students enrolled in blended Chinese as a second language (CSL) programs. Drawing on the L2 WTC framework, control-value theory, and self-efficacy theory, the study specified a theory-informed indirect-association model in which academic emotions and academic self-efficacy link motivation with communicative readiness. Survey data were collected from 956 international students in Chinese-language programs at eight universities in China. Measures covered learning motivation, positive academic emotions, negative academic emotions, academic self-efficacy, and L2 WTC. Confirmatory factor analysis and structural equation modeling with 5,000 bootstrap resamples were used to evaluate the measurement structure and the proposed indirect pathways. The structural model fitted the data well, *χ*^2^/df = 2.84, CFI = 0.96, TLI = 0.95, RMSEA = 0.044, and SRMR = 0.038. Learning motivation was directly associated with L2 WTC (*β* = 0.21, *p* < 0.001), and the total indirect association through emotions and self-efficacy (*β* = 0.34) was larger than the direct component. Positive emotions, lower negative emotions, and self-efficacy each accounted for part of the motivation-WTC association. Because the study used cross-sectional data, these pathways are interpreted as theoretically ordered associations rather than causal effects. The findings refine motivation-centered accounts of WTC by showing that motivation is linked with communicative readiness partly through emotionally supportive and capability-enhancing psychological conditions in blended CSL learning.

## 1 Introduction

A common difficulty in second language classrooms is the uneven relation between what learners know and what they are willing to do with that knowledge. Some students can complete written exercises, understand teacher explanations, and pass formal tests, yet remain silent when a spontaneous communicative opportunity appears. Others, even with incomplete linguistic control, enter interaction because the situation feels meaningful, manageable, and socially safe. Willingness to communicate (WTC) is useful because it names this final step between competence and use: the readiness to initiate discourse when a communicative possibility is available (MacIntyre et al., 1998; MacIntyre et al., 2001).

The construct has been widely studied, but the empirical base remains uneven. Much WTC research has been conducted in English-as-a-foreign-language or English-as-a-second-language settings. Chinese as a second language (CSL) deserves closer attention because it places learners in a different linguistic and social ecology. International students in China use Chinese across classroom tasks, campus services, online course platforms, peer relations, and daily problem-solving. They also work with a tonal system, character-based literacy, unfamiliar pragmatic conventions, and communication practices that may differ from those of alphabetic-language learning. Recent CSL research suggests that affective variables, motivational intensity, and self-beliefs are closely related to speaking performance and classroom participation beyond technology access or platform experience alone (Bao et al., 2026; Li, 2025; Sun et al., 2024).

The present study does not claim novelty merely from applying an existing WTC model to a Chinese-language program. Its central concern is theoretical: how motivational investment is associated with communicative readiness. Motivation is often treated as a positive antecedent of WTC, and meta-analytic evidence supports this general association (Elahi Shirvan et al., 2019). However, motivation does not automatically become speech, participation, or online interaction. Learners must evaluate whether the task matters, whether mistakes are tolerable, whether teachers and peers will respond supportively, and whether their current capability is sufficient for the situation. Control-value theory links value and control appraisals with achievement emotions (Pekrun, 2006; Pekrun, 2024; Pekrun et al., 2023). Self-efficacy theory adds that learners’ capability beliefs are closely related to whether they approach or avoid demanding action (Bandura, 1997). Together, these perspectives suggest that the motivation-WTC association may be organized through academic emotions and self-efficacy rather than through one undifferentiated motivational route.

Blended CSL learning adds a further layer to this problem. In this manuscript, blended learning is not used as a synonym for online learning. It refers to the integration of face-to-face classroom instruction with online components such as synchronous video meetings, learning-management-system activities, digital homework, asynchronous preparation, and course communication groups. This distinction is important because blended CSL programs distribute communication across classroom and digital spaces. Learners may prepare privately online, participate publicly in class, seek help through digital channels, and continue interaction in course groups. These arrangements may shape perceptions of value, control, social visibility, emotional risk, and capability.

This study examines a theory-specified indirect-association model using data from 956 international learners enrolled in blended Chinese-language programs at eight Chinese universities. The model distinguishes positive academic emotions, negative academic emotions, and academic self-efficacy as psychological conditions linking learning motivation with L2 WTC. The design is cross-sectional; therefore, the model does not establish temporal sequence or causal effects. Rather, it evaluates whether the observed covariance structure is consistent with a theoretically ordered account derived from WTC theory, control-value theory, and self-efficacy theory.

The study contributes to language-learning psychology in three ways. First, it tests the theoretical portability of a motivation-emotion-efficacy account in a large multinational CSL sample, rather than treating evidence from English-dominant L2 contexts as universally sufficient. Second, it refines motivation-centered explanations of WTC by asking whether academic emotions and academic self-efficacy account for part of the motivation-WTC association. Third, it conceptualizes blended CSL learning as a communicative ecology rather than as a technological delivery label, thereby shifting attention from platform access to the psychological organization of communication across online and face-to-face spaces.

## 2 Literature review and hypotheses

### 2.1 L2 WTC in Chinese-language learning

MacIntyre et al. (1998) placed WTC close to actual language behavior while allowing enduring and immediate conditions to shape it. The model is important because it does not reduce communication to proficiency. Topic, interlocutor, perceived competence, anxiety, desire to communicate, and social context can all alter whether a learner chooses to speak at a particular moment. Later work has confirmed the situated character of WTC across classroom tasks, out-of-class encounters, and digital environments (Pawlak and Mystkowska-Wiertelak, 2015; Zhang et al., 2018; Lee and Hsieh, 2019).

In CSL learning, WTC has a broader practical meaning than answering a teacher’s question. A learner in China may need to ask for academic help, negotiate group tasks, communicate with administrators, participate in online course forums, or manage everyday social contact. These situations vary in public visibility and perceived risk. The same learner may be willing to type a question in a class group but unwilling to speak in front of classmates, or may communicate confidently with peers while avoiding institutional interactions. This ecological variety makes CSL a useful context for examining psychological accounts of WTC.

Recent studies point in this direction. Work on CSL speaking performance suggests that affective factors can be more closely related to learning outcomes than technology-perception variables (Bao et al., 2026). Other studies connect CSL WTC with classroom emotions, grit, motivational intensity, and perceived competence (Li, 2025; Sun et al., 2024). These findings indicate that WTC should be studied as a psychological outcome situated within a specific educational ecology. The present study builds on this premise by asking whether motivation, academic emotions, and self-efficacy form a coherent explanatory pattern in blended CSL programs.

### 2.2 Learning motivation as a source of communicative orientation

Motivation gives learners reasons to remain invested in language study. From a self-determination perspective, some motives arise from interest, enjoyment, and personally endorsed value, whereas others are tied to academic achievement, professional plans, institutional requirements, or social mobility (Deci and Ryan, 1985; Ryan and Deci, 2017). In language learning, these motives are related to whether communication is interpreted as an opportunity for participation or as an unnecessary risk. A motivated learner may be more likely to look for chances to use the target language, tolerate errors, and treat difficult exchanges as information for improvement rather than as signs of failure.

The role of motivation may be especially visible in blended CSL courses. Learners must connect activities that occur across different spaces: listening to online materials, preparing assignments through digital systems, responding in face-to-face classes, and continuing interaction through course groups or learning platforms. Motivation may provide continuity across these spaces by helping learners see online preparation, classroom participation, and informal interaction as parts of the same learning project rather than as disconnected obligations.

Prior WTC research supports a positive association between motivation and communication. Meta-analytic evidence suggests that self-determined motivation is positively related to WTC (Elahi Shirvan et al., 2019). Studies of L2 motivational selves and affective pathways have also found that motivation is associated with WTC through enjoyment and anxiety (Sadoughi and Hejazi, 2024). In Chinese educational settings, learning motivation and academic self-efficacy have been linked with WTC in English-medium classrooms (Zhang and Dai, 2024). Interventions targeting confidence, anxiety, and achievement emotions can also alter engagement and communicative patterns (Ye and Hu, 2025; Yi et al., 2025).

At the same time, motivation should not be treated as communication itself. A learner may be motivated by examinations, scholarships, future employment, cultural interest, or family expectations without feeling ready to communicate in a specific classroom or online situation. The present model therefore retains the direct motivation-WTC pathway while examining whether motivation is also associated with WTC through academic emotions and self-efficacy.

*H1*: Learning motivation is positively associated with L2 willingness to communicate among international learners of Chinese.

### 2.3 Academic emotions as an affective pathway

Academic emotions are emotions attached to learning activities and achievement outcomes. They include enjoyment and pride as well as anxiety, boredom, anger, and hopelessness (Pekrun et al., 2011; Bieleke et al., 2021). Control-value theory argues that these emotions emerge from appraisals of value and control. When learners judge a task to be important and believe they can influence the outcome, positive activating emotions are more likely. When control is weak, value is unclear, or failure feels threatening, negative emotions become more salient (Pekrun, 2006; Pekrun, 2024).

Communication intensifies the emotional dimension of learning. Speaking Chinese in class or participating in an online group can expose pronunciation difficulties, lexical gaps, pragmatic uncertainty, or misunderstandings. Enjoyment and pride can make these risks more acceptable by framing communication as participation and growth. Anxiety, boredom, anger, or hopelessness can push learners away from interaction even when they generally value Chinese learning. Research on foreign language emotions has shown that enjoyment and anxiety are distinct constructs rather than opposite ends of a single continuum (Dewaele and MacIntyre, 2014; Wang et al., 2024).

Motivation is expected to be associated with these emotions through appraisal. Learners who see Chinese communication as meaningful and believe effort can produce progress are more likely to approach tasks with interest and pride. Learners with weaker perceived control may experience similar tasks as risky, tedious, or discouraging. Recent work has shown that enjoyment can account for relations between AI-assisted informal learning and L2 WTC (Guo and Wang, 2026), while research on online and blended learning links emotional engagement with academic outcomes (Liu et al., 2024). These findings make emotion a plausible affective pathway in the motivation-WTC association.

Positive and negative emotions are treated separately in the current model. This distinction matters pedagogically. Increasing enjoyment does not automatically remove anxiety, and reducing boredom does not necessarily create pride. A classroom may need meaningful topics and visible progress to support positive emotion, while also requiring clearer instructions, lower-stakes rehearsal, and supportive feedback to reduce negative emotion.

*H2a*: Learning motivation is positively associated with positive academic emotions.

*H2b*: Learning motivation is negatively associated with negative academic emotions.

### 2.4 Academic self-efficacy as a cognitive-evaluative pathway

Academic self-efficacy refers to learners’ beliefs that they can organize and carry out actions required for academic success (Bandura, 1997). In CSL learning, this belief includes confidence in understanding input, completing assignments, repairing mistakes, and communicating under classroom or online conditions. Within the WTC framework, perceived communicative competence and confidence are close to the point where a learner decides whether to communicate (MacIntyre et al., 1998). Academic self-efficacy is therefore a plausible bridge between general motivation and immediate communicative readiness.

Self-efficacy develops through mastery experience, vicarious experience, social persuasion, and physiological or affective states (Bandura, 1997). The emotional source is central in this study. Positive emotions can help learners read difficulty as manageable, broaden attention, and support expectations of future success (Fredrickson, 2001). Negative emotions may narrow attention, heighten threat perception, and make temporary difficulty feel like stable inability. In blended CSL learning, efficacy beliefs may also depend on whether learners can coordinate online preparation with live communication.

Recent work supports the relevance of self-efficacy to WTC in technology-enhanced and AI-mediated language contexts (Zhao et al., 2025; Tang et al., 2026). Tang et al. (2026), for instance, found that self-efficacy, grit, enjoyment, and anxiety were associated with willingness to communicate with generative AI tools. Although the present study focuses on human communication in Chinese, the underlying psychological logic is similar: learners are more willing to communicate when they believe they can manage both the linguistic task and the surrounding learning environment.

*H3a*: Positive academic emotions are positively associated with academic self-efficacy.

*H3b*: Negative academic emotions are negatively associated with academic self-efficacy.

*H4*: Academic self-efficacy is positively associated with L2 willingness to communicate.

### 2.5 Theory-specified serial indirect pathways in blended CSL learning

The proposed model is sequential in theoretical terms. Motivation gives Chinese learning value and direction. Through value and control appraisals, motivation is expected to be associated with how learners feel about learning activities and communicative tasks. These emotions then provide information about capability: enjoyment and pride can make participation feel possible, whereas anxiety, boredom, anger, or hopelessness may signal threat, low control, or limited competence. Self-efficacy becomes the proximal cognitive condition for WTC.

This sequence should not be read as evidence that all learners move through a fixed psychological script. Rather, it identifies a theoretically plausible ordering among constructs that previous theories have treated as interrelated. Control-value theory places appraisal and emotion between motivation and learning behavior (Pekrun, 2006; Pekrun, 2024). Self-efficacy theory explains why emotional experience is related to capability beliefs (Bandura, 1997). The WTC framework explains why those beliefs matter for communication (MacIntyre et al., 1998).

Because the present data are cross-sectional, serial indirect pathways are used as a theory-testing device rather than as proof of a temporal chain. Repeated successful communication may strengthen motivation; repeated anxiety may weaken motivation and efficacy. The present model should therefore be understood as a structured explanation of observed relations and as a basis for later longitudinal research.

Blended learning is treated as a meaningful context rather than as a causal variable. Online components can give learners time for preparation, but they can also reduce immediacy, create uncertainty about participation norms, and require additional self-regulation (Zhao and Song, 2022; Zhou et al., 2023). For international learners, online and face-to-face components may also differ in perceived safety. This context makes the emotion-efficacy organization of WTC especially important.

**Figure 1:**
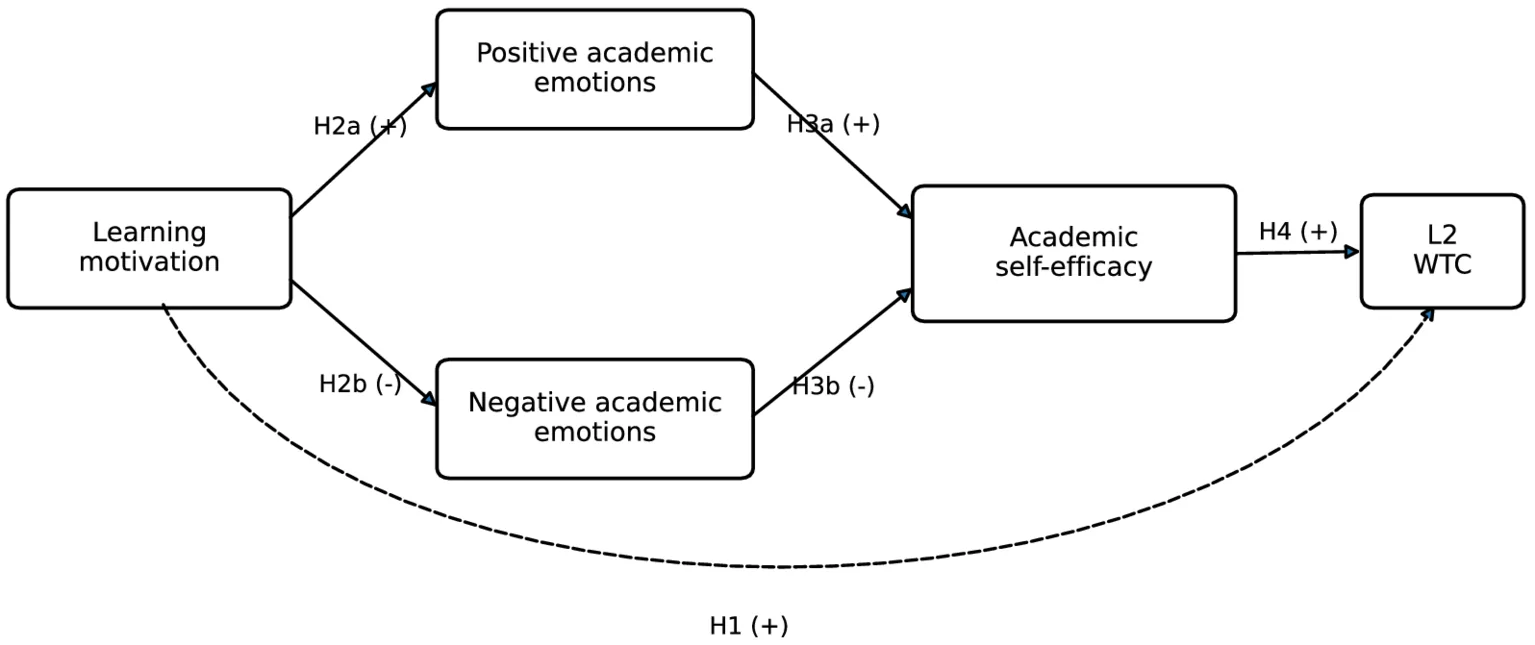
Hypothesized theory-specified indirect-association model.

*H5*: Academic emotions and academic self-efficacy form theory-specified serial indirect pathways in the association between learning motivation and L2 willingness to communicate (Figure 1).

## 3 Materials and methods

### 3.1 Research design and instructional context

This study employed a multi-institutional cross-sectional correlational survey design. The design was appropriate for examining whether the observed associations among learning motivation, academic emotions, academic self-efficacy, and L2 WTC were consistent with a theoretically specified model. However, because all focal variables were measured at one time point, the study cannot establish temporal precedence or causal effects. The structural paths reported below should therefore be interpreted as theoretically ordered associations rather than as evidence that one variable caused change in another.

The instructional setting was blended CSL learning. For the purposes of this study, a program counted as blended when regular face-to-face Chinese-language instruction was combined with at least one recurring online component used for course delivery, preparation, assignment submission, teacher-student communication, peer communication, or supplementary practice. The online component did not replace classroom instruction; rather, it formed part of an integrated learning environment in which learners prepared, practiced, submitted work, communicated with teachers and peers, and participated in classroom interaction.

Across the participating programs, the online components included combinations of synchronous video meetings, learning-management-system activities, digital homework submission, asynchronous preparation, and course communication groups. The exact combination of tools varied by institution and course. Blended learning is therefore treated in this study as the instructional ecology in which the psychological model was examined, not as a measured independent variable and not as an experimentally manipulated condition.

This operationalization is intentionally conservative. It allows the study to focus on learners who were studying Chinese through integrated face-to-face and online arrangements, while avoiding the unsupported claim that all participating programs used identical digital platforms, online intensity, or instructional design. Program-level variation in blended-learning implementation is addressed as a limitation below.

### 3.2 Participants and procedure

The analytic sample consisted of 956 international students enrolled in Chinese-language programs at eight universities in China during the 2023–2024 academic year. The universities were selected through access to intact CSL programs and professional contacts with instructors or program coordinators. The sampling design was therefore convenience-based rather than probability-based. This approach was suitable for reaching learners enrolled in actual blended CSL courses, but it limits institutional representativeness.

Recruitment occurred at both the program and student levels. Program coordinators or instructors were first contacted and asked whether they were willing to distribute the survey invitation to students enrolled in relevant Chinese-language courses. Institutional or instructor-level participation was voluntary. Students then received the survey link through course platforms or class communication groups. Student participation was also voluntary, and students were informed that choosing not to participate would not affect course grades, teacher evaluation, or access to learning resources.

Participants were eligible if they were international students enrolled in a Chinese as a second language course at one of the participating universities, were studying in a course that combined face-to-face instruction with online components as described above, were at least 18 years old, and provided informed consent. Responses were excluded if consent was not provided, if the respondent did not meet the instructional-context criterion, or if the questionnaire was substantially incomplete. No exclusion criterion was based on proficiency level, nationality, gender, or academic major.

Institutional identifiers were removed from the blinded manuscript and from the analytic file used for the current revision. For this reason, the manuscript does not report university names or city-level locations. This protects institutional anonymity but also limits the ability to model regional or university-level differences. The possible influence of institutional location, local language environment, program resources, and out-of-class communication opportunities is therefore acknowledged in the limitations section as an unmodeled source of variance.

Using course-based distribution allowed the study to reach learners who were participating in blended CSL instruction. It also means that the sample should be understood as an applied educational sample rather than a nationally representative probability sample. The inclusion of eight universities and 68 nationalities broadened the learner profile, but the study should not be interpreted as representing all CSL programs in China.

The sample included 524 female students (54.8%) and 432 male students (45.2%) from 68 countries. The largest country groups were South Korea (15.3%), Thailand (12.1%), Vietnam (9.7%), Russia (8.4%), and Pakistan (7.6%). Ages ranged from 18 to 42 years (*M* = 23.47, SD = 3.82). Regarding Chinese proficiency, 178 learners (18.6%) reported beginner proficiency corresponding to HSK 1–2, 432 (45.2%) reported intermediate proficiency corresponding to HSK 3–4, and 346 (36.2%) reported advanced proficiency corresponding to HSK 5–6. The mean length of Chinese study was 2.84 years (SD = 1.67) (Table 1).

**Table 1:** Participant profile in the analytic sample (*N* = 956).

Variable	Category	*n*	%
Gender	Female	524	54.8
	Male	432	45.2
Age group	18–22	387	40.5
	23–27	412	43.1
	28+	157	16.4
HSK level	Beginner (1–2)	178	18.6
	Intermediate (3–4)	432	45.2
	Advanced (5–6)	346	36.2
Study duration	< 1 year	186	19.5
	1–3 years	478	50.0
	> 3 years	292	30.5

Ethical procedures followed the local requirements for non-clinical educational survey research. The study did not involve clinical intervention, health-related manipulation, or collection of directly identifiable sensitive information. The questionnaire informed participants that demographic variables would be analyzed only in aggregate form and that responses would be used solely for research purposes.

### 3.3 Measures and instrument adaptation

All instruments were administered in Chinese because the study examined learners’ experience in Chinese-language programs. Bilingual glosses were available for lower-proficiency learners when a lexical item might otherwise affect comprehension. The wording of the constructs remained tied to Chinese learning, and the response format used a 5-point Likert scale from 1 (strongly disagree) to 5 (strongly agree) unless otherwise indicated.

A 5-point response format was used for three reasons. First, it is consistent with the response formats commonly used in educational survey research and with several source instruments or adapted versions used in L2 motivation, academic emotion, self-efficacy, and WTC research. Second, the sample included learners at different Chinese proficiency levels; a 5-point format was judged to reduce response burden and make the distinction among response options easier for lower-proficiency participants than a more fine-grained 6- or 7-point format. Third, a 5-point format retained a neutral midpoint, which was appropriate because some learners might genuinely feel uncertain or mixed about their emotions, self-efficacy, or willingness to communicate in blended CSL situations.

The use of a 5-point scale also has methodological implications. Fewer response categories may reduce variability relative to 7-point formats, whereas a midpoint may increase central-tendency responding. To address this concern, the revised manuscript reports reliability and latent-variable validity evidence rather than relying on response format alone. The SEM results were interpreted cautiously as associations among latent constructs, and the response format is acknowledged as part of the measurement context rather than as evidence of validity by itself.

Because the study used established instruments in a CSL and blended-learning context, the adaptation process addressed both linguistic clarity and construct relevance. The procedure involved four stages. First, the source instruments were reviewed against the theoretical definitions of the five focal constructs: learning motivation, positive academic emotions, negative academic emotions, academic self-efficacy, and L2 WTC. Items were retained or adapted only when their content corresponded to the construct definition used in the present study. Second, the wording was contextualized for Chinese-language learning and, where necessary, for blended learning situations involving both classroom and online communication. Third, specialists in language education and educational psychology reviewed the adapted items for content coverage, construct relevance, and contextual appropriateness. Fourth, a pilot check with CSL learners of similar backgrounds was conducted to examine item clarity, response burden, and potential misunderstandings.

The validation process was therefore not limited to language mechanics. It was intended to evaluate whether the adapted items adequately represented the intended constructs in the CSL context. Because a formal content validity index was not part of the original design, the manuscript does not claim quantitative CVI evidence. Instead, content relevance was addressed qualitatively through expert review and pilot checking, and the formal sample provided psychometric evidence through CFA, internal consistency, composite reliability, convergent validity, and discriminant validity.

Learning motivation was assessed with an adapted Academic Motivation Scale (Vallerand et al., 1992). The version used in this study contained 20 items representing intrinsic motivation, extrinsic motivation, and amotivation. Intrinsic items referred to interest, enjoyment, and personal value in learning Chinese. Extrinsic items referred to academic, professional, and instrumental reasons. Amotivation items were reverse scored before scale construction so that higher total scores represented stronger overall motivation. Internal consistency was strong for the total scale (*α* = 0.91), and subscale reliabilities ranged from 0.82 to 0.89.

Academic emotions were measured with a shortened Achievement Emotions Questionnaire adapted for second language learning (Pekrun et al., 2011). The 24 items represented enjoyment, pride, anxiety, boredom, anger, and hopelessness. Consistent with control-value theory and prior language-emotion research, enjoyment and pride were modeled as positive academic emotions, whereas anxiety, boredom, anger, and hopelessness were modeled as negative academic emotions. Reliability coefficients ranged from 0.81 to 0.90 across the subscales; internal consistency coefficients for the positive- and negative-emotion composite scores were 0.88 and 0.89.

Academic self-efficacy was assessed with a 12-item Chinese-learning self-efficacy scale adapted from the self-efficacy subscale of the Motivated Strategies for Learning Questionnaire (Pintrich et al., 1991). Items asked learners to report confidence in understanding course content, completing Chinese-learning tasks, achieving academic success in Chinese courses, and managing communicative demands in blended learning. Cronbach’s alpha was 0.93.

L2 willingness to communicate was assessed with a 12-item scale adapted from MacIntyre et al. (2001). Items addressed willingness to use Chinese in classroom discussion, out-of-class interaction, reading-related exchanges, and writing-related communicative situations. Because the study focused on blended Chinese learning, the wording included communicative opportunities across both face-to-face and online course activities. Cronbach’s alpha was 0.92.

Scale scores were computed so that higher values represented stronger endorsement of the relevant construct. Negative emotion items were not reverse scored into a positive index; instead, negative academic emotions were retained as a distinct latent variable. This decision preserved the theoretical difference between experiencing more positive emotion and experiencing less negative emotion.

Parcel indicators were created from theoretical subdimensions rather than from purely empirical item groupings. The motivation parcels reflected intrinsic motivation, extrinsic motivation, and reverse-scored amotivation. The positive-emotion parcels reflected enjoyment and pride. The negative-emotion parcels grouped the negative emotion subdimensions into two conceptually interpretable parcels to maintain a stable indicator-to-sample-size ratio. The academic self-efficacy and WTC parcels were formed to represent the major content domains covered by their scales. Parceling was used to estimate relations among latent constructs rather than item-level dimensionality. At the same time, because parceling can mask item-level misfit and inflate apparent model fit, this decision is reported transparently and treated as a measurement limitation.

### 3.4 Data analysis

The analysis proceeded in four steps. First, the data were screened for missing values, univariate distributions, common method bias, and multicollinearity. Missing-data patterns were examined before model estimation. Full information maximum likelihood was used because missingness was low and did not show evidence of systematic departure from randomness. Skewness and kurtosis were inspected to assess univariate distribution. Harman’s single-factor test was used only as an initial diagnostic for common method bias, and variance inflation factors were inspected for multicollinearity.

Second, descriptive statistics and Pearson correlations were computed for learning motivation, positive academic emotions, negative academic emotions, academic self-efficacy, and L2 WTC. Third, confirmatory factor analysis evaluated the latent measurement structure. The model specified five latent constructs matching the theoretical model. The measurement evidence was evaluated using standardized factor loadings, Cronbach’s alpha, composite reliability, average variance extracted, and discriminant-validity evidence.

Fourth, structural equation modeling examined whether the observed covariance structure was consistent with the hypothesized theoretical model. Model fit and robust parameter estimates were evaluated using MLR. Bias-corrected confidence intervals for indirect effects were obtained in a separate ML analysis with 5,000 bootstrap draws (Preacher and Hayes, 2008). Because the design was cross-sectional, the model was not interpreted as demonstrating causal mediation or temporal change.

Model fit was evaluated using multiple indices rather than a single global criterion. Following widely used SEM guidelines, *χ*^2^/df values below about 3, CFI and TLI values at or above 0.90 were interpreted as acceptable and values near 0.95 as stronger evidence of fit; RMSEA values below 0.08 were interpreted as acceptable and values near or below 0.06 as stronger evidence; SRMR values below 0.08 were interpreted as acceptable. These cutoffs were used as interpretive guidelines, not rigid rules, because model fit also depends on sample size, model complexity, distributional assumptions, and theoretical plausibility (Hu and Bentler, 1999; Hair et al., 2019).

The final structural model was theory-driven. Mplus modification indices were not used to add *post hoc* paths, free correlated residuals, or alter the substantive interpretation of the model. The tested model included theoretically defensible direct associations from motivation to WTC, from motivation to self-efficacy, and from academic emotions to WTC, because a strict full-serial-mediation model would have been overly restrictive and would have forced all emotion-WTC associations to pass through self-efficacy. These direct paths were interpreted as partial-mediation controls rather than as post hoc empirical modifications.

Students were nested within eight universities, which means that observations may not have been fully independent. Ideally, institution-level identifiers would allow intraclass correlations, cluster-robust standard errors, or a multilevel SEM robustness check. However, university identifiers were removed from the anonymized analytic file used for the current revision, so clustering could not be modeled. The potential non-independence of observations is therefore acknowledged as a design limitation, and the findings are interpreted at the learner level rather than as institution-level effects.

## 4 Results

### 4.1 Preliminary analyses and correlations

Missing data were low. No focal variable had more than 3% missing values. Little’s MCAR test did not indicate a non-random missingness pattern, *χ*^2^ = 124.37, df = 118, *p* = 0.33. Full information maximum likelihood was therefore used to retain all available information. Skewness ranged from −0.67 to 0.54, and kurtosis ranged from −0.48 to 0.71, supporting acceptable univariate distribution for the focal variables. Harman’s single-factor test extracted 12 factors with eigenvalues above 1.0; the first factor accounted for 28.4% of the variance, below the commonly used 40% diagnostic threshold. Variance inflation factors were below 3.0, suggesting that multicollinearity was not problematic.

Table 2 presents means, standard deviations, and zero-order correlations. The correlation pattern supported the expected direction of the model before latent-variable testing. Learning motivation was positively associated with positive academic emotions, academic self-efficacy, and L2 WTC, and negatively associated with negative academic emotions. Positive academic emotions were positively associated with academic self-efficacy and WTC. Negative academic emotions had negative associations with self-efficacy and WTC.

**Table 2:** Means, standard deviations, and zero-order associations among focal variables (*N* = 956).

Variable	*M*	SD	1	2	3	4	5
1. LM	3.84	0.62	–				
2. PAE	3.71	0.68	0.56***	–			
3. NAE	2.43	0.71	−0.38***	−0.41***	–		
4. ASE	3.52	0.73	0.51***	0.54***	−0.36***	–	
5. L2 WTC	3.46	0.76	0.47***	0.49***	−0.34***	0.53***	–

LM, Learning motivation; PAE, Positive academic emotions; NAE, Negative academic emotions; ASE, Academic self-efficacy; L2 WTC, Second language willingness to communicate. ***p < 0.001.

The bivariate associations also helped rule out a trivial explanation for the model. If motivation, emotions, self-efficacy, and WTC had been only weakly related, the proposed sequence would have had little empirical basis. Instead, the correlations showed a coherent pattern: motivation was linked to more favorable emotional experience and stronger efficacy beliefs, and both were connected with greater communicative readiness.

### 4.2 Measurement model

The CFA specified five latent variables: learning motivation, positive academic emotions, negative academic emotions, academic self-efficacy, and L2 WTC. The model showed a good fit to the data: *χ*^2^(67) = 189.42, *p* < 0.001, *χ*^2^/df = 2.83, CFI = 0.97, TLI = 0.96, RMSEA = 0.044, 90% CI [0.036, 0.051], and SRMR = 0.032. This pattern indicated that the proposed latent structure represented the data adequately.

The measurement results are important because the model combines constructs that are conceptually related but not identical. Motivation, positive emotion, self-efficacy, and WTC all contain positive psychological content, which can sometimes produce inflated associations in survey data. The evidence for discriminant validity indicates that the constructs retained separable empirical meanings within this sample.

All standardized factor loadings were above 0.70. Composite reliability calculated from the displayed loadings ranged from 0.79 to 0.87, and average variance extracted ranged from 0.60 to 0.69. For each construct, the square root of AVE was higher than its correlations with the other constructs, supporting discriminant validity. Table 3 summarizes the CFA evidence and provides the basis for testing the structural pathways.

**Table 3:** Latent-variable measurement evidence from confirmatory factor analysis.

Construct/indicator	λ	CR	AVE	√AVE
Learning motivation		0.83	0.63	0.79
LM parcel 1	0.84			
LM parcel 2	0.81			
LM parcel 3	0.72			
Positive academic emotions		0.79	0.65	0.81
PAE parcel 1	0.86			
PAE parcel 2	0.75			
Negative academic emotions		0.81	0.68	0.82
NAE parcel 1	0.88			
NAE parcel 2	0.76			
Academic self-efficacy		0.87	0.69	0.83
ASE parcel 1	0.91			
ASE parcel 2	0.85			
ASE parcel 3	0.73			
L2 WTC		0.86	0.60	0.77
WTC parcel 1	0.82			
WTC parcel 2	0.79			
WTC parcel 3	0.76			
WTC parcel 4	0.72			

λ, Standardized factor loading; CR, Composite reliability; AVE, Average variance extracted; √AVE, Square root of AVE.

### 4.3 Structural model

The structural model showed a good fit to the data: *χ*^2^(71) = 201.64, *p* < 0.001, *χ*^2^/df = 2.84, CFI = 0.96, TLI = 0.95, RMSEA = 0.044, 90% CI [0.037, 0.052], and SRMR = 0.038. The direct pathway from learning motivation to L2 WTC remained significant after the intervening variables were included, *β* = 0.21, SE = 0.04, *p* < 0.001, consistent with H1. Motivation was positively associated with positive academic emotions, *β* = 0.52, SE = 0.03, *p* < 0.001, and negatively associated with negative academic emotions, *β* = −0.35, SE = 0.04, *p* < 0.001; these paths were consistent with H2a and H2b.

The emotion-to-efficacy paths were also significant. Positive academic emotions were associated with higher academic self-efficacy, *β* = 0.38, SE = 0.04, *p* < 0.001, whereas negative academic emotions were associated with lower self-efficacy, *β* = −0.19, SE = 0.04, *p* < 0.001. These results were consistent with H3a and H3b. Academic self-efficacy was positively associated with L2 WTC, *β* = 0.41, SE = 0.05, *p* < 0.001, consistent with H4. Additional paths showed that motivation was directly associated with self-efficacy, *β* = 0.24, SE = 0.04, *p* < 0.001; positive academic emotions were associated with WTC, *β* = 0.17, SE = 0.04, *p* < 0.001; and negative academic emotions were associated with lower WTC, *β* = −0.12, SE = 0.04, *p* = 0.003. These direct emotion and efficacy paths were retained to avoid attributing the full motivation-WTC relation to the serial chain alone.

**Figure 2:**
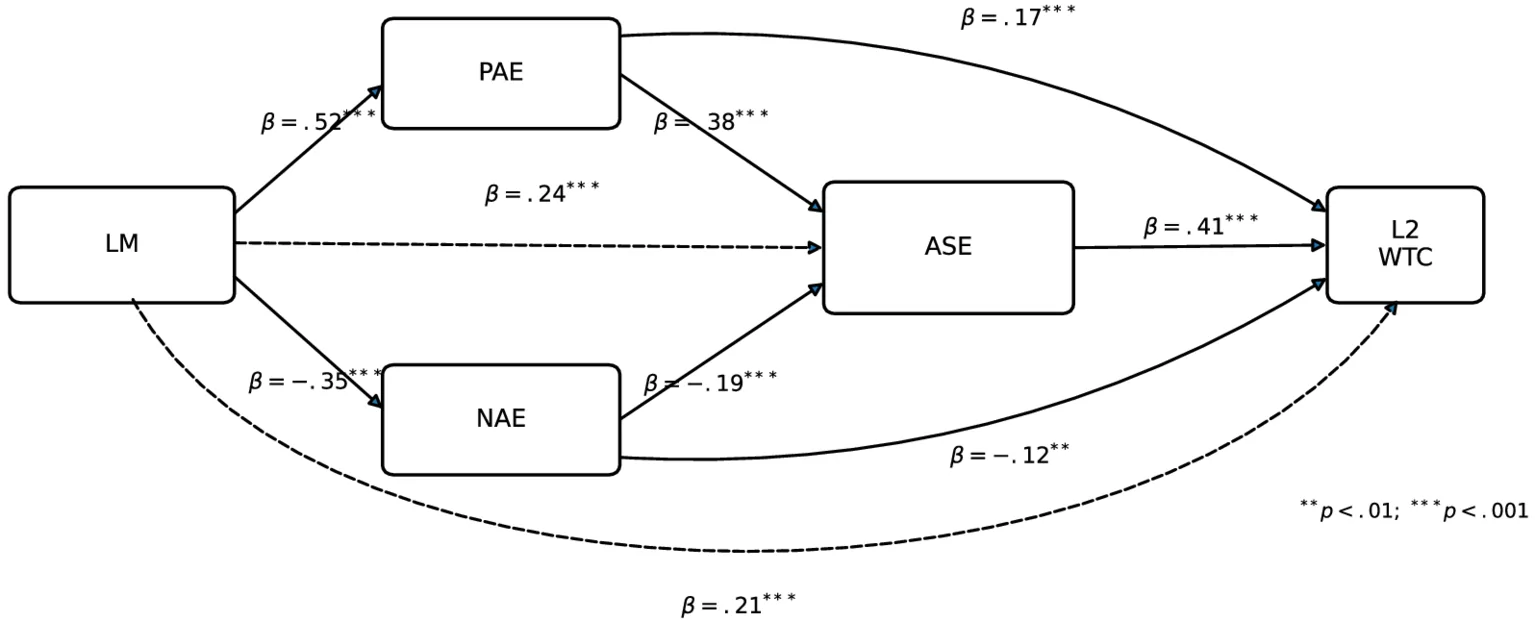
Standardized estimates in the tested structural model.

This modeling decision was specified on theoretical grounds rather than derived from *post hoc* modification indices. A pure serial model would have forced all emotion associations to pass through self-efficacy. The retained direct associations recognize that emotions may also be related to WTC in ways not fully captured by efficacy beliefs. For example, enjoyment may make communication attractive even before a learner consciously evaluates competence, while anxiety may suppress participation even when competence beliefs are reasonably strong. The results should nevertheless be read as associations within one theoretically preferred model, not as evidence that alternative orderings are impossible (Figure 2; Table 4).

**Table 4:** Standardized estimates for the structural paths.

Path	*β*	SE	*p*	Interpretation
LM - > L2 WTC	0.21	0.04	< 0.001	Consistent with H1
LM - > PAE	0.52	0.03	< 0.001	Consistent with H2a
LM - > NAE	−0.35	0.04	< 0.001	Consistent with H2b
PAE - > ASE	0.38	0.04	< 0.001	Consistent with H3a
NAE - > ASE	−0.19	0.04	< 0.001	Consistent with H3b
LM - > ASE	0.24	0.04	< 0.001	Additional direct association
PAE - > L2 WTC	0.17	0.04	< 0.001	Additional direct association
NAE - > L2 WTC	−0.12	0.04	0.003	Additional direct association
ASE - > L2 WTC	0.41	0.05	< 0.001	Consistent with H4

### 4.4 Indirect associations

The standardized coefficients reported in Table 4 imply five indirect associations: through positive academic emotions (*β* ≈ 0.09), through academic self-efficacy (*β* ≈ 0.10), through the serial sequence of positive academic emotions and academic self-efficacy (*β* ≈ 0.08), through lower negative academic emotions (*β* ≈ 0.04), and through lower negative academic emotions followed by stronger self-efficacy (*β* ≈ 0.03). Their sum is approximately 0.34. Together with the direct association of 0.21, the implied total association is approximately 0.55; the indirect component represents approximately 61.6% of the total. This pattern is consistent with H5.

**Figure 3:**
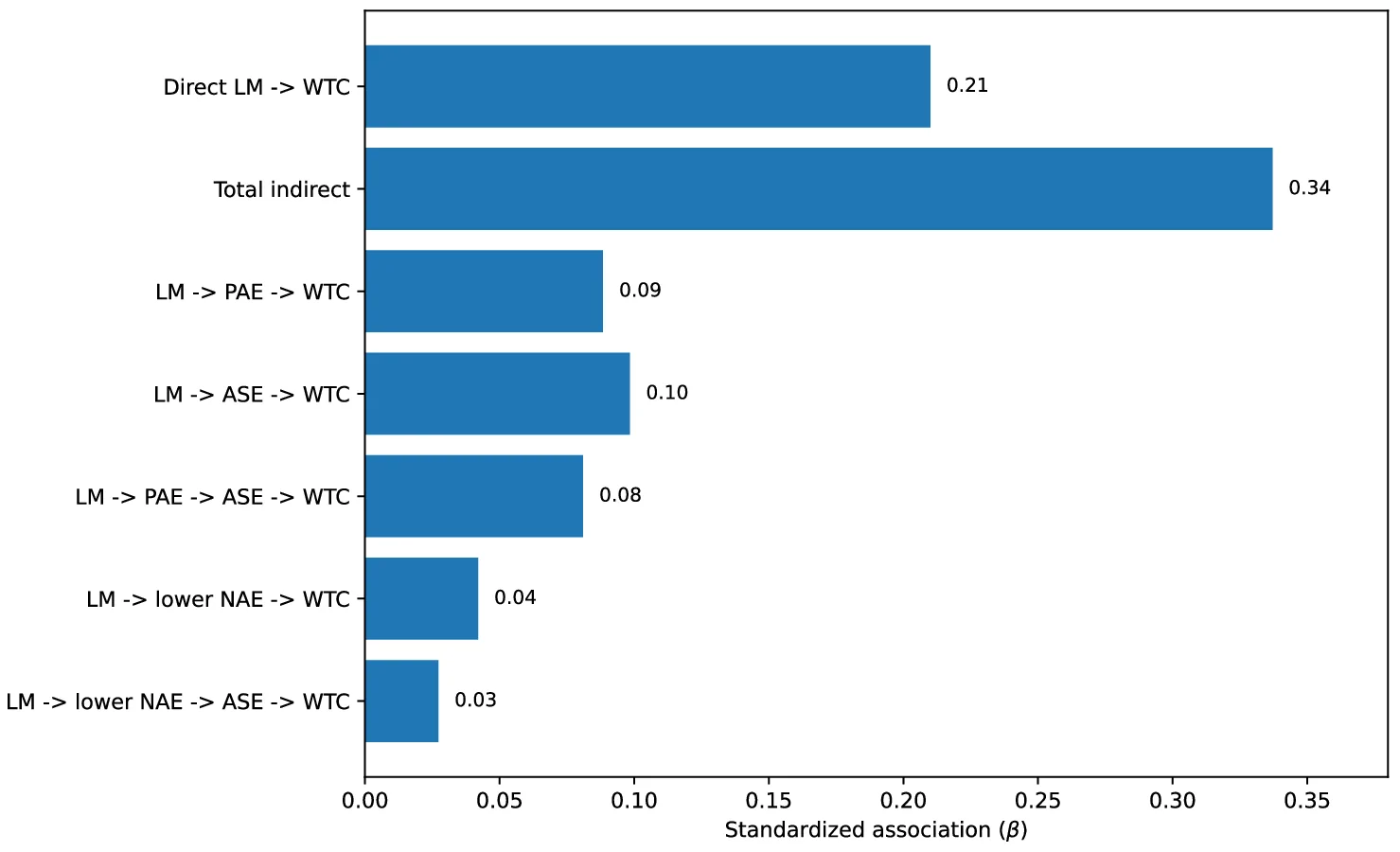
Direct and indirect associations as standardized coefficients.

The size of the indirect associations also supports a nuanced interpretation. No single pathway dominated the model to the point of making the others redundant. Instead, motivation appeared to be linked to WTC through several smaller but theoretically coherent channels. This pattern is compatible with the view that communication emerges from converging psychological resources rather than from one isolated factor (Figure 3; Table 5).

**Table 5:** Decomposition of standardized direct and indirect associations.

Association/pathway	*β*	% of total
Total standardized association	0.55	100.0
Direct component (LM - > WTC)	0.21	38.39
Total indirect component	0.34	61.61
Ind1: LM - > PAE - > WTC	0.09	16.16
Ind2: LM - > ASE - > WTC	0.10	17.99
Ind3: LM - > PAE - > ASE - > WTC	0.08	14.81
Ind4: LM - > lower NAE - > WTC	0.04	7.68
Ind5: LM - > lower NAE - > ASE - > WTC	0.03	4.98

Values are calculated from the standardized coefficients reported in Table 4 and are approximate. Percentages may not sum exactly because of rounding.

## 5 Discussion

### 5.1 Motivation is related to communication through supportive psychological conditions

The main purpose of this study was to clarify how learning motivation is associated with WTC among international learners of Chinese in blended programs. The results show that motivation mattered, but not in the simple sense of directly moving learners into communication. The direct path from motivation to WTC was significant, yet the combined indirect component was larger. This pattern suggests that learners’ motivation is linked to communication partly through the emotional quality of learning and the belief that communication is within reach.

This interpretation is consistent with self-determination theory while adding explanatory detail from control-value theory and self-efficacy theory. A learner may value Chinese for academic progress, employment, intercultural identity, or personal interest. However, such value may still fail to correspond to participation when the classroom feels threatening or when the learner doubts their ability to manage an exchange. Motivation therefore needs psychological support. The findings align with studies linking motivational orientations, emotions, and WTC in language learning (Sadoughi and Hejazi, 2024; Zhang and Dai, 2024; Guo and Wang, 2026), while extending that work to a large CSL sample.

For CSL learners in China, the mechanism has practical relevance. Chinese is encountered not only in language classes but also in administrative offices, online learning platforms, peer networks, group projects, and everyday problem-solving. A learner may be motivated to study Chinese for future plans but still hesitate when speaking to classmates, writing to teachers, or participating in an online course group. The findings indicate that blended Chinese courses should not treat motivation as a fixed learner trait. Teachers can help connect motivation with communication by making success visible, normalizing error, and designing participation routines that are emotionally safe.

This finding also speaks to how teachers interpret silence. Silence in a CSL classroom may be mistaken for lack of motivation, resistance, or insufficient preparation. The present model suggests other possibilities. A learner may be motivated but anxious, interested but unsure of capability, or confident online but hesitant in face-to-face interaction. Pedagogical responses should therefore diagnose the psychological condition behind non-participation rather than assuming a single cause.

### 5.2 Academic emotions operate as a hinge between value and participation

The emotional findings are central to the argument. Learning motivation was associated with stronger positive emotions and weaker negative emotions. These emotions were in turn associated with both self-efficacy and WTC. This pattern fits control-value theory: learners who value Chinese learning and perceive greater control are more likely to experience enjoyment and pride, whereas learners with lower control or unclear value are more vulnerable to anxiety, boredom, anger, or hopelessness (Pekrun, 2006; Pekrun, 2024).

The results also show why blended learning needs psychological evaluation. Online components can give learners access to materials and time for rehearsal, but their value depends on how they are experienced. If a platform produces confusion or isolation, communication may decline even when resources are available. If online tasks allow learners to prepare, receive feedback, and see progress, they can support positive emotion and participation. Bao et al. (2026) similarly found that affective factors were more closely related to Chinese speaking performance than platform-perception variables. The current results extend that insight to WTC.

The separation of positive and negative emotions is pedagogically useful. Enjoyment and pride may help learners approach communication, but reducing anxiety or boredom is a different task. A course that creates interesting topics may still leave students anxious if evaluation is public and unforgiving. Conversely, a course that reduces pressure may not generate engagement if tasks feel mechanical. Emotion-sensitive blended design should therefore cultivate positive emotional energy while reducing avoidable emotional barriers.

This distinction can guide teacher intervention. To foster positive emotion, teachers can use personally relevant topics, collaborative tasks, and visible markers of progress. To reduce negative emotion, they can clarify expectations, provide rehearsal time, reduce unnecessary public evaluation, and frame errors as resources for learning. A blended design can support both goals when online preparation is connected to classroom use instead of being treated as separate homework.

### 5.3 Self-efficacy is the closest psychological bridge to WTC

Academic self-efficacy was the strongest proximal correlate of L2 WTC in the structural model. This result accords with Bandura’s (1997) argument that efficacy beliefs are linked with choice and persistence, and with MacIntyre et al.’s (1998) placement of communicative confidence near the behavioral point of L2 communication. In the present study, learners who believed they could manage Chinese academic and communicative demands were more willing to communicate.

The serial indirect pathway clarifies how self-efficacy is associated with emotion. Positive emotions were related to stronger efficacy beliefs, while negative emotions were related to weaker efficacy beliefs. In other words, emotion was not just a background reaction; it provided information that learners could use when judging their own capability. Enjoyment and pride may make Chinese communication feel possible and rewarding. Anxiety, boredom, anger, or hopelessness may make the same task feel risky or unmanageable.

The result also suggests that efficacy-building should occur before high-stakes communication. If learners meet difficult public tasks before they have accumulated manageable successes, they may interpret struggle as evidence that they cannot communicate. If they first experience partial mastery through guided rehearsal, peer modeling, and feedback, later communication may feel more attainable.

In blended CSL teaching, efficacy support should be deliberate. Online preparation can strengthen mastery when it includes short rehearsal activities, model responses, diagnostic feedback, and progress records. Classroom communication can then turn private preparation into public use. Teachers can also support efficacy by giving feedback that names what learners can already do, explains why errors are repairable, and provides next steps.

### 5.4 Contributions to language-learning psychology

The findings refine a motivation-centered interpretation of L2 WTC. A common assumption in WTC research is that more motivated learners are more likely to communicate. The present results do not reject this assumption, but they make it more conditional. Motivation was directly associated with L2 WTC, yet a substantial part of the association was linked to academic emotions and academic self-efficacy. This suggests that motivation should not be treated as a self-sufficient explanation of communicative readiness. A learner may value Chinese learning and still avoid communication if the task is emotionally threatening or if the learner doubts their ability to manage the interaction.

The study also refines affective explanations of WTC by distinguishing positive and negative academic emotions. Positive emotions and negative emotions were not treated as opposite ends of a single continuum. This distinction is theoretically important because enjoyment and pride may support approach-oriented participation, whereas anxiety, boredom, anger, or hopelessness may inhibit communication through different psychological routes. The findings suggest that increasing positive emotion and reducing negative emotion are related but non-identical pedagogical tasks.

A further contribution concerns the interpretation of blended learning. The results should not be read as evidence that blended learning causes WTC. Rather, they suggest that blended CSL learning provides an ecology in which motivational, emotional, and efficacy-related processes become especially visible. Learners move between private online preparation, public classroom participation, digital communication with teachers and peers, and out-of-class Chinese use. These movements may intensify questions of value, control, social visibility, and perceived capability. The study therefore shifts the discussion of blended CSL learning away from a technology-access perspective and toward a psychological-process perspective.

The contribution is also methodological. The large sample permitted estimation of a model that included both positive and negative academic emotions. Keeping these emotion categories separate avoids a common problem in which affect is treated as a single broad factor. The results show that positive and negative emotions are not merely mirror images. They provide different intervention targets and different explanatory routes to WTC.

At the same time, the cross-sectional design requires caution. The structural model is consistent with the proposed theoretical ordering, but it cannot establish that motivation temporally precedes emotion, that emotion causes changes in self-efficacy, or that self-efficacy causes later WTC. The findings should therefore be understood as evidence of theoretically coherent associations that warrant further longitudinal, diary-based, and experimental investigation.

### 5.5 Practical implications

The findings have practical implications for teachers, curriculum designers, and CSL program administrators. For teachers, the main implication is that increasing motivation alone is unlikely to be sufficient. Communicative tasks should be designed to create visible progress, positive emotional experience, and manageable risk. For example, instructors can begin a topic with private online preparation, move to pair rehearsal, then small-group exchange, and only later invite whole-class communication. This sequence allows learners to accumulate partial success before communication becomes publicly visible.

Teachers can also use low-stakes communication routines that connect directly with learners’ everyday needs in China. Examples include asking for help from an instructor, explaining an absence, negotiating group-project roles, discussing campus services, sending a message in a class group, making a short appointment request, or responding to a peer’s question. These tasks are pedagogically useful because they connect motivation with real communicative purposes while reducing the gap between classroom Chinese and daily Chinese use.

Feedback should be designed to support self-efficacy rather than only to correct linguistic form. Instead of marking errors as final failures, teachers can identify what the learner successfully communicated, explain which repair strategy would improve the message, and provide a next-step task that is achievable within a short time. Feedback such as ‘your request is clear, but the tone marker needs repair; try this version and send it again’ is more likely to build perceived capability than feedback that only lists mistakes.

For curriculum designers, the results suggest that blended CSL courses should align online and face-to-face components around communicative readiness. Online tasks should prepare learners for later classroom use, not simply add homework. For example, an online listening or vocabulary task can be followed by a classroom information-gap activity; a written forum post can become the basis for oral discussion; and digital rehearsal recordings can be used for self-reflection before live speaking.

For program administrators, the findings point to the importance of institutional conditions that make communication possible beyond individual classrooms. Programs can provide structured Chinese-use opportunities such as peer language partners, guided campus-service simulations, low-pressure conversation corners, and online help channels moderated by trained tutors. Such supports may be especially useful for learners who are motivated but hesitant because of anxiety or low self-efficacy.

Finally, blended-learning design should be monitored from a psychological as well as technological perspective. Access to a platform does not guarantee emotional support or communicative readiness. Programs should examine whether online components clarify expectations, provide rehearsal opportunities, offer timely feedback, and connect with face-to-face communication. If online tasks are fragmented or disconnected from classroom interaction, they may add workload without strengthening WTC.

### 5.6 Limitations and future research

Several limitations require caution. First, the data were cross-sectional. Although the model was theoretically ordered and tested through indirect-association analysis, causal claims cannot be made. Longitudinal, diary-based, and experimental designs are needed to examine whether changes in motivation precede changes in emotions, efficacy beliefs, and WTC over time.

Second, alternative models remain plausible. Self-efficacy may shape learners’ emotional experiences, WTC may strengthen later motivation through successful communication, and motivation, emotions, self-efficacy, and WTC may influence one another reciprocally over time. The present model is theoretically preferred because it integrates WTC theory, control-value theory, and self-efficacy theory, but the cross-sectional design cannot rule out competing temporal orderings. Future research should compare alternative models using longitudinal or experimental data.

Third, students were nested within eight universities. Learners from the same university or program may share similar teachers, digital platforms, curriculum structures, institutional resources, peer environments, or out-of-class opportunities to use Chinese. Because institution-level identifiers were not available in the anonymized analytic file used for the current revision, intraclass correlations, cluster-robust standard errors, and multilevel SEM could not be estimated. This means that possible clustering effects remain an unmodeled source of variance.

Fourth, the participating universities were accessed through intact programs and professional contacts rather than through probability sampling. Institutional location may matter because different regions of China can offer different local language environments, demographic profiles, program resources, and opportunities for out-of-class communication. Exact institutional and city-level identifiers were not reported in the blinded manuscript, so the findings should be interpreted as evidence from a multi-institutional applied sample rather than as nationally representative evidence.

Fifth, blended learning was operationalized at the program level. The study identified learners as participating in courses that integrated face-to-face instruction with online components, but it did not measure the intensity, quality, platform type, or pedagogical integration of each online component. Therefore, the findings should not be interpreted as showing that blended learning itself produces higher WTC. Future studies should measure specific blended-learning features, such as online preparation, synchronous discussion, asynchronous forums, teacher feedback, peer interaction, and digital rehearsal, and examine how each is related to emotion, self-efficacy, and WTC.

Sixth, the study relied on self-report measures. Reliability and validity evidence supported the measurement model, but self-report data can be influenced by social desirability, response style, central-tendency responding, and learners’ interpretations of scale items. Future research should include classroom observation, communication logs, digital participation traces, peer ratings, or teacher ratings. Behavioral indicators would help determine whether reported WTC is linked with actual communication.

Seventh, the study used a 5-point Likert response format. This format reduced response burden for a linguistically diverse sample, but it may also have constrained response variability compared with more fine-grained formats. Future studies could compare 5-point and 7-point formats or test measurement invariance across proficiency levels to examine whether response format affects reliability and latent-construct interpretation.

Eighth, parcel indicators were used in the CFA and SEM. The parcels were constructed from theoretical subdimensions, but parceling can mask item-level multidimensionality, suppress localized misfit, and improve apparent model fit. Future research should report item-level CFA or supplemental item-level measurement models before parceling, especially when adapting instruments across languages and educational contexts.

Ninth, the study did not include direct behavioral measures of communication. WTC is theoretically close to behavior, but it is still a self-reported readiness state. Future studies should examine whether the same emotion-efficacy pattern is related to actual speaking turns, message posts, help-seeking episodes, peer interaction, or teacher-rated participation.

Finally, the study used positive and negative emotion composites. This decision supported model parsimony, but individual emotions may have different associations. Enjoyment, pride, anxiety, boredom, anger, and hopelessness may not follow identical pathways. Future work should examine emotion-specific processes and test whether particular emotions are more strongly linked to self-efficacy or WTC.

## 6 Conclusion

This study examined how learning motivation is associated with L2 willingness to communicate among 956 international learners of Chinese in blended CSL contexts. The findings support a theory-specified indirect-association account: motivation was associated with academic emotions, emotions were associated with academic self-efficacy, and self-efficacy was associated with L2 WTC. More than half of the total motivation-WTC association was represented by indirect pathways.

The theoretical message is that motivation alone is not enough to explain communicative readiness. In blended CSL learning, motivated learners may become more willing to communicate when learning conditions are emotionally sustainable and when learners believe they can manage the task. Because the data are cross-sectional and institution-level clustering was not modeled, this conclusion should be read as a theory-informed account of associations rather than as causal evidence.

For research on international Chinese education, the study highlights the need to move beyond enrollment growth or proficiency outcomes alone. Communicative participation depends on how learners experience the learning environment psychologically. A learner’s willingness to use Chinese is shaped not only by what they know, but also by whether they feel that participation has value, whether it is emotionally tolerable, and whether they believe they can manage the task.

## Data Availability

The data analyzed in this study are not publicly available. Further inquiries may be directed to the corresponding author.
